# RareCure: An Open-Source Artificial Intelligence Pipeline for Context-Adaptive Treatment Discovery in Rare Solid Tumors

**DOI:** 10.7759/cureus.109744

**Published:** 2026-05-27

**Authors:** Danielmartin Arogyasami

**Affiliations:** 1 Computational Precision Oncology, Independent Researcher, Carmel, USA

**Keywords:** artificial intelligence, clinical trial matching, investigational decision support, open source, precision oncology, rare cancer, sarcoma

## Abstract

Background

Rare cancers account for approximately 25-30% of cancer diagnoses in the United States, yet precision-oncology infrastructure has been built primarily for common tumor types. Soft tissue sarcomas exemplify this national gap: over 50 histological subtypes affect approximately 13,000 Americans each year, fewer than 5% of subtypes have dedicated clinical trials, and five-year metastatic survival has remained below 20% for decades. The National Cancer Institute has identified acceleration of precision approaches for understudied cancers as a federal strategic priority. This work introduces RareCure, an open-source artificial intelligence pipeline that automatically generates evidence-ranked therapeutic option dossiers for rare solid tumors, released under the Massachusetts Institute of Technology (MIT) license for unrestricted adoption by United States academic medical centers, community oncology practices, and resource-limited research settings.

Methods

RareCure integrates six computational modules previously available only as disconnected tools: somatic variant processing with tiered annotation; neoantigen prediction with dual human leukocyte antigen (HLA) resolution (architectural; batch-scale validation pending); drug-gene matching across four curated databases with harmonized scoring; clinical trial screening with ontology-aware query expansion to surface basket trials invisible to rare-subtype searches; retrieval-augmented evidence generation; and a context-adaptive orchestration agent using large language model (LLM) reasoning constrained by deterministic weight clamping, functionally verified through boundary condition testing. The pipeline was validated retrospectively on 260 soft tissue sarcoma patients from The Cancer Genome Atlas Sarcoma cohort (TCGA-SARC). A dual deployment architecture supports cloud-hosted LLMs for de-identified research data and locally hosted open-source models for institutional settings under the Health Insurance Portability and Accountability Act (HIPAA).

Results

The pipeline executed end-to-end on all 260 patients. At least one Tier 1 or Tier 2 drug match (US FDA-approved or genomically matched) was identified in 30.0% of patients (78/260; 95% confidence interval (CI): 24.5-36.0%), consistent with the 20-40% range reported in independent sarcoma genomic profiling studies, with partial overlap noted between the TCGA cohort and the OncoKB knowledge base used for tier annotation. Biomarker-driven matching was achieved in 78.8% of patients (205/260; 95% CI: 73.4-83.6%). Interpretation cost was $303.74 ($1.17 per patient), excluding upstream sequencing. Deterministic weight clamping triggered in 0.0% of standard runs; a boundary condition test confirmed correct interception of extreme weight distributions.

Conclusions

RareCure demonstrates that end-to-end treatment discovery for rare solid tumors can be automated within a single open-source pipeline, producing actionability rates concordant with published benchmarks at interpretation costs compatible with broad research applicability. The deterministic clamping design pattern, adaptive LLM reasoning within auditable bounds, has applicability beyond oncology to clinical artificial intelligence requiring regulatory traceability. Module-level ablation and external cohort validation are designated next steps. Source code is freely available under the MIT license without licensing barriers.

## Introduction

Rare cancers, defined by the European Society for Medical Oncology as those with an incidence below six per 100,000 annually, collectively account for approximately 25-30% of all cancer diagnoses in the United States and Europe [1]. Soft tissue sarcomas (STS) alone affect approximately 13,000 Americans each year [1]. Despite this collective burden, individual rare subtypes receive disproportionately less research investment and are underrepresented in clinical trial portfolios [2]. Soft tissue sarcomas (STS) exemplify this disparity: the category encompasses over 50 histologically distinct subtypes recognized by the World Health Organization classification [3], with fewer than 5% of subtypes having dedicated clinical trials and many lacking subtype-specific clinical trial data entirely [4]. Five-year overall survival for metastatic STS has remained below 20% for decades [5], and for subtypes including adult osteosarcoma and spindle cell sarcoma, first-line protocols have not substantively changed since the 1980s.

This treatment gap represents a nationally significant healthcare challenge. Cancer remains the second leading cause of death in the United States, and the National Cancer Institute has identified the acceleration of precision approaches for understudied cancers--including rare subtypes--as a federal strategic priority. The broader precision health vision articulated across federal initiatives depends on computational tools that can translate genomic data into actionable treatment options at scale. For the majority of rare cancer patients, however, no such tool exists. When standard options are exhausted, patients enter a treatment void characterized by limited trial availability, long wait times for molecular tumor board review, and reliance on individual physician knowledge to identify off-label or experimental options [6]. Recent cases demonstrate that a fundamentally different approach is feasible. In 2024-2025, Sid Sijbrandij, co-founder of GitLab, assembled a dedicated research team employing comprehensive multi-omic sequencing, AI-assisted interpretation, and more than ten parallel experimental therapies--including fibroblast activation protein (FAP)-targeted radioligand therapy, personalized messenger RNA (mRNA) neoantigen vaccines, and checkpoint inhibitors--to achieve no evidence of disease from recurrent osteosarcoma [7]. The estimated cost exceeded one million U.S. dollars. Separately, Australian engineer Paul Conyngham used large language models (LLMs) and AlphaFold to design a personalized mRNA vaccine for his dog's mast cell cancer, achieving approximately 75% tumor shrinkage at a sequencing cost of approximately $3,000 (reported in Fortune and The Conversation, March 2026). These are existence proofs, but neither approach scales to the broader patient population.

Several resources address individual pipeline components, but none integrate them into an end-to-end system that produces a complete, evidence-ranked option dossier. OncoKB provides variant annotation but does not match patients to trials [8]. The Drug-Gene Interaction Database (DGIdb) catalogs drug-gene relationships but requires manual interpretation [9]. pVACtools provides neoantigen prediction but operates standalone [10]. ClinicalTrials.gov requires manual keyword searching [11]. Large-scale clinical genomic profiling studies have demonstrated the value of comprehensive sequencing [12], yet the interpretation bottleneck--translating genomic findings into ranked treatment options--remains largely manual. Notably, existing tools produce context-independent output: identical results for a given variant regardless of whether the patient is newly diagnosed or has exhausted five lines of therapy [13].

This study presents RareCure with two methodological contributions: ontology-aware clinical trial matching that expands rare subtype queries through a cancer type hierarchy to surface basket trials and tumor-agnostic studies; and context-adaptive ranking in which an LLM dynamically generates scoring weights based on disease acuity, constrained by deterministic clamping within clinically defined bounds for auditability and functionally verified through boundary condition testing. The key contributions of this work are fourfold. First, a unified pipeline was designed that integrates variant annotation, neoantigen prediction, drug-gene matching, clinical trial screening, literature evidence synthesis, and context-adaptive ranking--capabilities that currently exist only as disconnected tools requiring manual coordination. Second, a hierarchical query expansion strategy was developed that surfaces basket trials and tumor-agnostic studies invisible to direct rare-subtype searches, addressing the fundamental discovery barrier for uncommon histologies. Third, an orchestration approach was designed in which LLM-generated scoring weights are constrained by deterministic clamping, providing adaptive reasoning within auditable bounds--a design pattern applicable beyond oncology to any clinical AI system requiring both flexibility and regulatory traceability. Fourth, the complete pipeline was validated on 260 sarcoma patients from a public dataset, and all source code, synthetic test data, and containerized deployment were released under the Massachusetts Institute of Technology (MIT) license to enable reproducibility and adoption by the broader research community.

Within this scope, the present study evaluates two co-primary endpoints: end-to-end execution feasibility across the full The Cancer Genome Atlas Sarcoma cohort (TCGA-SARC) and the Tier 1/2 actionability rate benchmarked against published sarcoma genomic profiling literature. Demonstration of clinical utility is explicitly out of scope and is reserved for future prospective work.

Module-level ablation analysis isolating the marginal contributions of ontology expansion, multi-database harmonization, and context-adaptive scoring was not performed in this study and is designated as the primary validation gap to be addressed by future work. This work is intended for research purposes only and is not designed, validated, or approved for clinical use, diagnosis, or treatment of any medical condition.

In practical terms, a user of RareCure submits either a somatic variant file (mutation annotation format (MAF) or variant call format (VCF), with optional human leukocyte antigen (HLA) typing) or a structured clinical profile describing histology, stage, prior treatments, and biomarkers. The pipeline automatically annotates variants for clinical actionability, queries four drug-gene databases in parallel, performs ontology-aware searches of ClinicalTrials.gov, retrieves supporting literature from an indexed PubMed corpus, and synthesizes the combined evidence into an evidence-ranked dossier of investigational therapeutic options. Each option is annotated with its evidence tier, mechanism, and regulatory access pathway--standard prescription, clinical trial, off-label with National Comprehensive Cancer Network (NCCN) compendium support, or compassionate use-- and the dossier is structured for review by a qualified clinician rather than for direct prescriptive use.

## Materials and methods

System architecture and deployment modes

**Figure 1:**
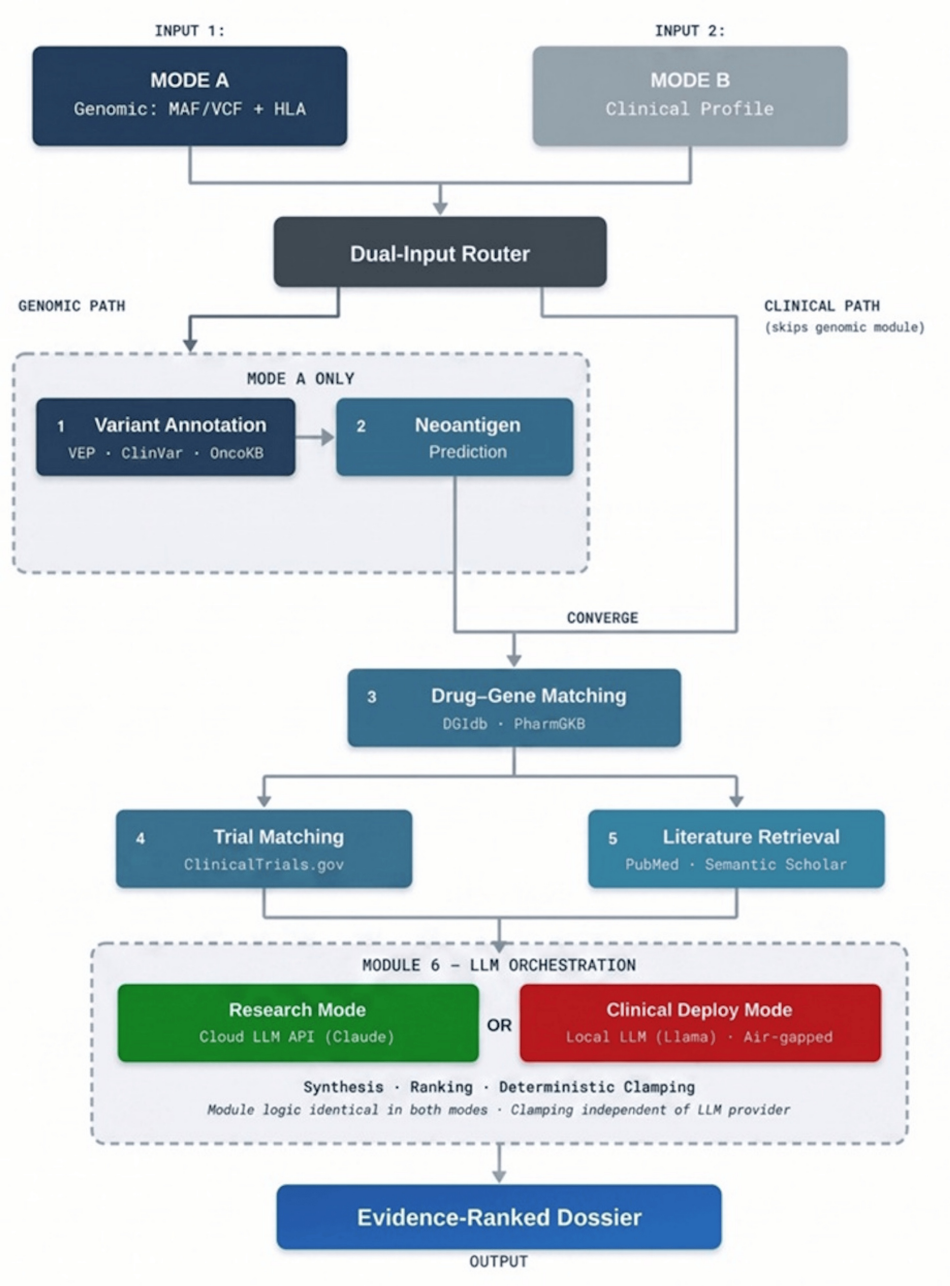
System architecture. Six-module pipeline architecture and dual deployment modes. Mode A accepts genomic input (mutation annotation format (MAF) or variant call format (VCF) with optional human leukocyte antigen (HLA) typing); Mode B accepts a structured clinical profile and bypasses Modules 1 and 2. Module 6 orchestrates synthesis and ranking via an LLM and applies deterministic weight clamping; clamping operates independently of the LLM provider. Research mode connects to a cloud-hosted LLM via application programming interface (API) for analysis of de-identified public data; clinical deployment mode uses a locally hosted open-source LLM for environments subject to the Health Insurance Portability and Accountability Act (HIPAA) or other privacy regulations, with no patient data leaving the local environment. Module logic, interfaces, and scoring methodology are identical in both modes; only the LLM endpoint differs. Image generated using Microsoft PowerPoint. API: application programming interface; HIPAA: Health Insurance Portability and Accountability Act; HLA: human leukocyte antigen; LLM: large language model; MAF: mutation annotation format; PHI: protected health information; VCF: variant call format; VEP: variant effect predictor.

RareCure was designed as a six-module pipeline implemented in Python 3.12 (Python Software Foundation, Wilmington, Delaware, USA) (Figure 1), with a model-agnostic architecture that decouples research validation from clinical deployment requirements. The pipeline accepts two input modes: Mode A (genomic: mutation annotation format (MAF) or variant call format (VCF) with optional RNA expression and HLA typing) and Mode B (clinical: structured profile with histological subtype, stage, metastases, prior treatments, biomarkers, demographics, and geography). Modules 1 and 2 require genomic input and are bypassed in Mode B.

The orchestration agent employs the Reasoning and Acting (ReAct) paradigm [14], in which the LLM alternates between generating natural-language reasoning traces and invoking tools that return structured observations. In RareCure's implementation, each tool corresponds to one of Modules 1 through 5: the agent produces an interim reasoning step (for example, "Tier 2 CDK4 amplification detected; query trial registry under dedifferentiated liposarcoma and broader parents"), dispatches the corresponding module call, incorporates the returned evidence into its working context, and proceeds to the next reasoning step. Reasoning traces, tool invocations, and intermediate observations are persisted for audit. The ReAct loop terminates when all planned module calls have resolved, at which point the orchestrator issues a final structured synthesis request with the accumulated context. The implementation follows the reference pattern [14] without architectural modification. The architecture supports two deployment configurations via an LLM_PROVIDER parameter. For research mode, using de-identified public datasets (such as TCGA), the pipeline connects to a cloud-hosted LLM via application programming interface (API). All experiments reported in this study used Claude 3.5 Sonnet (Anthropic, San Francisco, CA, USA) in this mode. Because TCGA data are fully de-identified and publicly accessible under the National Institutes of Health Genomic Data Sharing Policy, no protected health information (PHI) was transmitted to external endpoints.

For clinical deployment mode, involving patient data subject to the Health Insurance Portability and Accountability Act (HIPAA), the General Data Protection Regulation (GDPR), or other privacy regulations, the pipeline operates with a locally hosted open-source LLM (for example, Llama 3.1-70B, Meta AI, Menlo Park, CA, USA) running entirely within the institution's network. In this configuration, no patient data leaves the local environment. The LLM_PROVIDER parameter switches between modes without any change to the pipeline logic, module interfaces, or scoring methodology.

Modules 3 and 4 employ asynchronous hypertext transfer protocol (HTTP) input/output via httpx with semaphore-based rate limiting (10 concurrent requests). API responses are cached in a thread-safe time-to-live cache (threading.Lock on all mutations, one-hour expiry, 2000-entry maximum).

Source code, synthetic test data for reproducibility, and a Docker container are available under the MIT license [15]. The system described herein is a research prototype and has not been evaluated or cleared by the U.S. Food and Drug Administration or any other regulatory body.

Data sources and cohort

Masked somatic mutation data (MAF) and clinical annotations for STS patients were obtained from TCGA-SARC via the National Cancer Institute Genomic Data Commons [16]. The TCGA-SARC cohort comprises specimens collected between 2006 and 2013 from multiple contributing institutions [16]. After excluding patients with missing essential annotations (defined as absent histological subtype or zero reported mutations), 260 patients spanning multiple histological subtypes were retained, including leiomyosarcoma, undifferentiated pleomorphic sarcoma, dedifferentiated liposarcoma, myxofibrosarcoma, synovial sarcoma, malignant peripheral nerve sheath tumor, and others (Table 1).

**Table 1:** Baseline clinical characteristics of the TCGA-SARC validation cohort (N = 260). Liposarcoma counts aggregate dedifferentiated, well-differentiated, and pleomorphic subtypes. Percentages are calculated against the full 260-patient cohort and may not sum to 100% due to rounding. TCGA-SARC: The Cancer Genome Atlas Sarcoma Cohort.

Characteristics	Value
Leiomyosarcoma	95 (36.5%)
Liposarcoma	59 (22.7%)
Undifferentiated pleomorphic sarcoma	30 (11.5%)
Fibromyxosarcoma	21 (8.1%)
Malignant fibrous histiocytoma	13 (5.0%)
Synovial sarcoma	10 (3.8%)
Malignant peripheral nerve sheath tumor	8 (3.1%)
Other	24 (9.2%)

Per-patient HLA class I allele calls were obtained from Thorsson et al. [17], who derived alleles using OptiType [18], cross-referenced with Shukla et al. [19] to identify somatic HLA loss-of-heterozygosity. Drug-gene interactions were queried from DGIdb v4.0 [9], CIViC [20], OncoKB (public tier) [8], and ChEMBL [21]. Clinical trials were queried from the ClinicalTrials.gov REST API v2 [11]. Biomedical literature was obtained via the National Center for Biotechnology Information (NCBI) File Transfer Protocol (FTP) baseline Extensible Markup Language (XML) datasets, filtered to sarcoma, neoantigen, and radioligand therapy publications, and indexed between January 2020 and the January 2026 baseline release, in English language only.

Module 1: Somatic variant processing

A four-tier evidence classification system was designed to map somatic variants to clinical actionability, drawing on the tiering frameworks established by OncoKB [8] and CIViC [20] but harmonizing them into a unified hierarchy suitable for multi-source integration. This four-tier approach was selected because no existing tool provides a single harmonized evidence classification spanning OncoKB, CIViC, and the Catalogue Of Somatic Mutations In Cancer (COSMIC) simultaneously.

MAF files were parsed using a chunked reading strategy (100,000-row segments) implemented to handle multi-gigabyte files without exceeding memory constraints--a practical consideration absent from many existing variant processing tools that assume pre-filtered inputs. Variants were filtered to coding mutations and classified into four evidence tiers. Tier 1 (known actionable) comprises variants with OncoKB Level 1/2/R1 or CIViC Level A evidence with matching cancer type. Tier 2 (likely actionable) comprises variants with OncoKB Level 3A/3B, CIViC Level B, or hotspot mutations (COSMIC recurrence of 5 or more) [22]. Tier 3 (uncertain significance) comprises variants predicted deleterious (Sorting Intolerant From Tolerant (SIFT) score < 0.05 [23] or Polymorphism Phenotyping v2 (PolyPhen-2) score > 0.85 [24]) without clinical evidence. Tier 4 (likely passenger) comprises variants with benign or tolerated predictions and no cancer gene relevance.

Patients with zero retained coding variants were routed to clinical-only mode with a recommendation for fusion-detection assays. In clinical-only mode, Modules 1 and 2 are bypassed and Module 3 performs drug-gene matching against a curated set of frequently mutated genes for the patient's histological subtype, derived from published genomic profiling studies [6] and National Comprehensive Cancer Network (NCCN)-recognized molecular targets [4]. This fallback ensures that patients without individual sequencing data still receive subtype-informed options grounded in the peer-reviewed literature rather than generic pan-cancer recommendations.

Module 2: Neoantigen prediction with dual human leukocyte antigen (HLA) resolution

A dual-resolution neoantigen prediction strategy was designed to address both genomic-mode and clinical-mode inputs within a single architectural framework. Mode A uses patient-specific HLA-A/B/C alleles for personalized binding predictions via pVACtools [10] with NetMHCpan 4.1 [25] and MHCflurry 2.0 [26]. Mode B generates predictions against seven high-frequency supertypes: HLA-A*02:01, HLA-A*01:01, HLA-A*03:01, HLA-A*24:02, HLA-B*07:02, HLA-B*08:01, and HLA-B*44:02 [27,28]. All Mode B outputs carry a mandatory warning flag. Candidates are filtered by binding affinity below 500 nM, differential agretopicity index above 1.0 [29], and variant allele frequency (VAF) at or above 0.05. Neoantigen prediction validation at the batch scale is designated as future work.

Module 3: Drug-gene interaction matching

A concurrent multi-source querying strategy was designed to address the central challenge of drug-gene matching for rare tumors: no single database provides comprehensive coverage. By querying four databases in parallel--DGIdb [9], CIViC [20], OncoKB [8], and ChEMBL [21]--and harmonizing results through the four-tier system described above, the pipeline captures interactions that would be missed by any individual source. For each gene harboring Tier 1-3 variants, all four databases were queried concurrently via httpx.AsyncClient with asyncio. Semaphore. Results were deduplicated by drug-gene pair, harmonized into the four-tier system with the highest evidence tier retained for conflicts, and all conflicts logged for audit. The OncoKB annotated variants database was downloaded once into local memory, eliminating per-patient API calls and reducing latency.

Module 4: Clinical trial matching with ontology expansion

**Figure 2:**
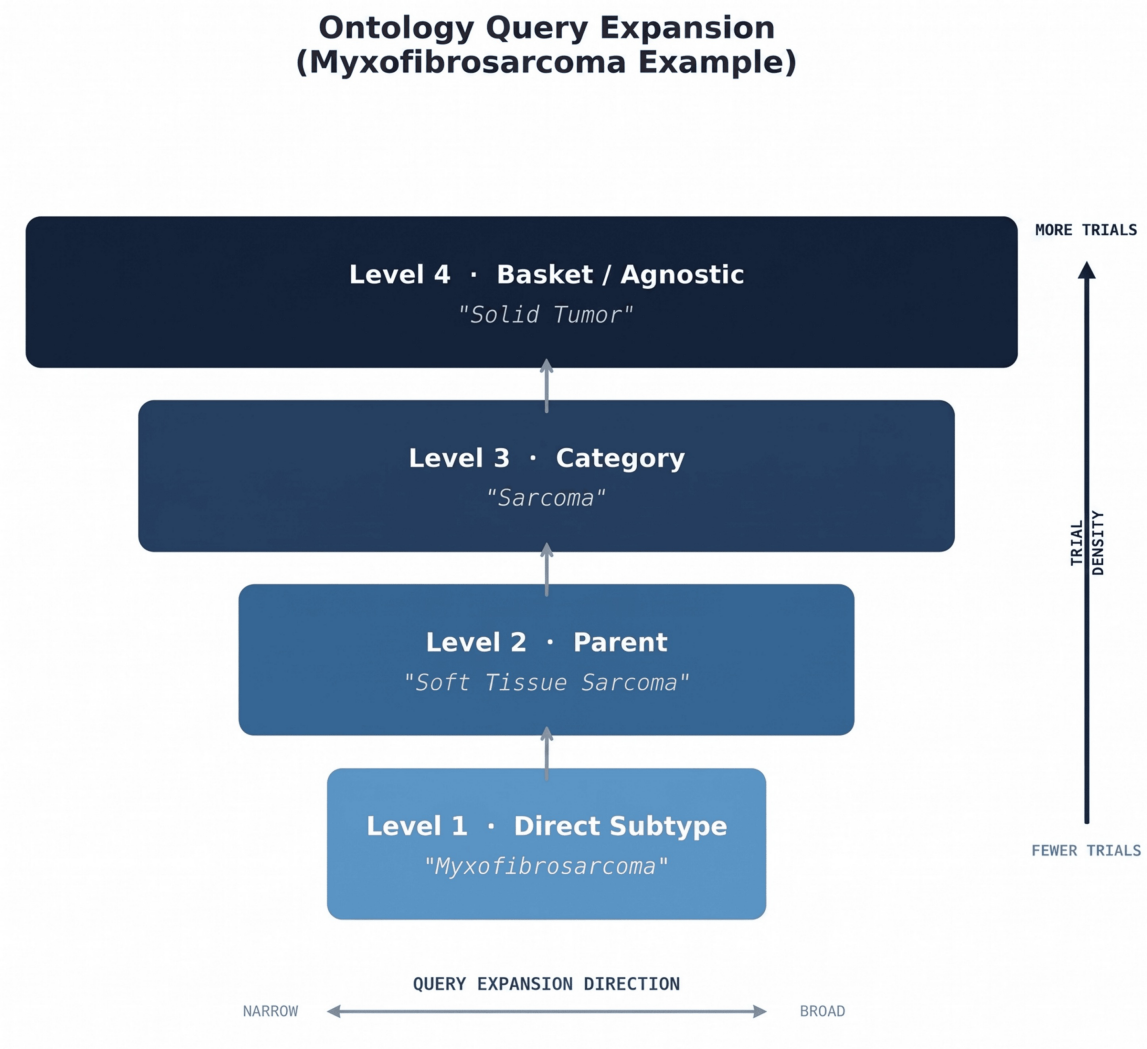
Ontology query expansion (myxofibrosarcoma example). Hierarchical ontology-aware query expansion strategy illustrated using myxofibrosarcoma as the input subtype. The pipeline progressively broadens each ClinicalTrials.gov query across four levels: the specific histological subtype (level 1, narrowest), the parent histology (level 2, soft tissue sarcoma), the histological category (level 3, sarcoma), and basket or tumor-agnostic trials (level 4, solid tumor, broadest). All four queries are executed concurrently rather than sequentially, ensuring that basket trials and tumor-agnostic studies invisible to direct rare-subtype keyword searches are surfaced alongside any subtype-specific trials. Shading intensity indicates expected trial density at each level (darker = more trials available). Representative expansion paths for additional sarcoma subtypes are shown in Table 2. Image generated using Microsoft PowerPoint.

An ontology-aware query expansion strategy was designed to overcome the fundamental discovery barrier for rare tumor subtypes: direct subtype queries against ClinicalTrials.gov return few or zero results because most trials recruit under broader histological categories. Queries were expanded using a cancer type hierarchy (Figure 2)--for example, "spindle cell sarcoma" → "soft tissue sarcoma" → "sarcoma" → "solid tumor"--thereby surfacing basket trials and tumor-agnostic studies invisible to direct keyword matching. This hierarchy covers 11 sarcoma subtypes with a default fallback. All expanded queries were executed concurrently. Representative expansion paths are shown in Table 2.

At each expansion level, the query set grows cumulatively: a search for myxofibrosarcoma generates four concurrent queries, one per level, ensuring that Phase III basket trials recruiting "solid tumor" patients and Phase II studies recruiting "sarcoma" patients are surfaced alongside any subtype-specific trials. Relevance scoring weighted genomic match (0.35), trial phase (0.30), histology match (0.20), and geographic proximity (0.15). Genomic match was weighted highest because molecular evidence of targetability provides the strongest signal for rare tumors where histology-specific trial data may be absent. These weights were selected by the author to operationalize NCCN's emphasis on molecular target concordance as the primary driver of trial eligibility for sarcoma patients [4], and are exposed as configuration parameters so that adopting institutions can re-calibrate them against local molecular tumor board precedent. Formal derivation through structured expert elicitation (for example, a Delphi panel) and weight-sensitivity analysis are designated as future work.

**Table 2:** Ontology expansion paths for representative sarcoma subtypes. At each expansion level, the query set grows cumulatively, with one ClinicalTrials.gov query dispatched per level. All four queries are executed concurrently rather than sequentially. MPNST: malignant peripheral nerve sheath tumor.

Input subtype	Level 1 (direct)	Level 2 (parent)	Level 3 (category)	Level 4 (basket/agnostic)
Myxofibrosarcoma	Myxofibrosarcoma	Soft tissue sarcoma	Sarcoma	Solid tumor
Spindle cell sarcoma	Spindle cell sarcoma	Soft tissue sarcoma	Sarcoma	Solid tumor
Synovial sarcoma	Synovial sarcoma	Soft tissue sarcoma	Sarcoma	Solid tumor
Dedifferentiated liposarcoma	Dedifferentiated liposarcoma	Liposarcoma	Sarcoma	Solid tumor
Leiomyosarcoma	Leiomyosarcoma	Soft tissue sarcoma	Sarcoma	Solid tumor
MPNST	Malignant peripheral nerve sheath tumor	Soft tissue sarcoma	Sarcoma	Solid tumor

Module 5: Retrieval-augmented evidence generation

NCBI FTP baseline distribution was selected over sequential E-utilities queries to enable offline operation and avoid per-request rate limits--a design decision motivated by the clinical deployment requirement for air-gapped environments with no external network access. PubMed abstracts obtained via NCBI FTP baseline were chunked at 512 tokens--selected through preliminary experiments comparing 256, 512, and 1024-token windows, where 512 tokens preserved paragraph-level context without exceeding embedding model input limits-- and embedded with all-MiniLM-L6-v2 [30], then stored in ChromaDB (Chroma, San Francisco, California, United States). The top five chunks by cosine similarity (threshold 0.65, selected empirically to balance precision and recall across sarcoma query terms) were passed to the LLM for structured evidence summaries. To mitigate hallucination risk, the LLM was instructed to provide PubMed identifiers (PMIDs) for all therapeutic claims, which were programmatically cross-referenced against the retrieved context chunks; any claim citing a PMID absent from the retrieval set was flagged as unsupported and excluded from the final evidence summary. This citation-grounding mechanism ensures that all surfaced evidence is traceable to indexed biomedical literature rather than LLM-generated assertions.

Module 6: Context-adaptive orchestration with deterministic clamping

The orchestration module was designed around a central insight: for rare cancers, context must influence option ranking, but unconstrained AI reasoning introduces unacceptable variability for clinical use. To address this tension, a deterministic clamping mechanism was developed that permits LLM-guided weight generation while enforcing bounds derived from clinical reasoning.

The patient context is passed to the LLM with a few-shot prompt containing two reference examples (full prompt template, including the system message, few-shot examples, and expected JavaScript Object Notation (JSON) schema, is available as prompt_template.md in the public repository cited above). Weights are generated five times at a temperature of 0.3, and the element-wise median is taken. Each weight is then clamped within clinically informed bounds (evidence strength: 0.05-0.60; access feasibility: 0.05-0.40; expected response: 0.05-0.50; safety profile: 0.03-0.40; cost: 0.02-0.35), then re-normalized. The output carries a clamped boolean for audit tracking. These bounds were selected by the author to reflect two principles: first, that no single scoring dimension should dominate the composite score (hence the ceilings), and second, that no clinically relevant dimension should be effectively zeroed out by adaptive reasoning (hence the floors). The specific values were chosen to approximate the range of considerations described in published molecular tumor-board practice [6,31] rather than being derived from a formal expert elicitation exercise, and are exposed as version-controlled configuration parameters so that adopting institutions can tighten or relax them against local governance requirements. Formal derivation of bounds through structured expert elicitation and sensitivity analysis is designated as future work.

The composite score is the minimum of 1.0 and the product of the sum of weighted components and a novelty bonus factor (1.0 plus 0.15 if the mechanism is novel). Failed prior therapies are excluded.

To verify the clamping mechanism's functional engagement at the edge of its operating envelope, a boundary condition test was designed. An extreme clinical scenario was constructed ("patient demands zero toxicity, safety is the only consideration"), intended to force a degenerate weight distribution (safety = 1.0, all other dimensions = 0.0). The clamping mechanism correctly intercepted the extreme output, forcing safety from 1.0 to 0.40 (the configured upper bound) and redistributing weight across the remaining dimensions within their respective bounds. This confirms that the mechanism engages as designed under at least one constructed boundary condition; systematic perturbation-based evaluation across a structured suite of edge cases is designated as future work.

Statistical analysis

Proportions were calculated with Clopper-Pearson 95% confidence intervals. All analyses were performed in Python 3.12 using the SciPy library. The significance threshold was 0.05. Exact library versions are pinned in the project's requirements.txt file and the published Docker image to ensure computational reproducibility.

## Results

The Cancer Genome Atlas Sarcoma cohort (TCGA-SARC) retrospective analysis

The pipeline was validated on the TCGA-SARC cohort. Of 261 TCGA-SARC patients, 260 met the inclusion criteria spanning multiple sarcoma subtypes, with leiomyosarcoma, undifferentiated pleomorphic sarcoma, and dedifferentiated liposarcoma as the three largest subgroups. Baseline subtype distribution is shown in Table 1.

**Figure 3:**
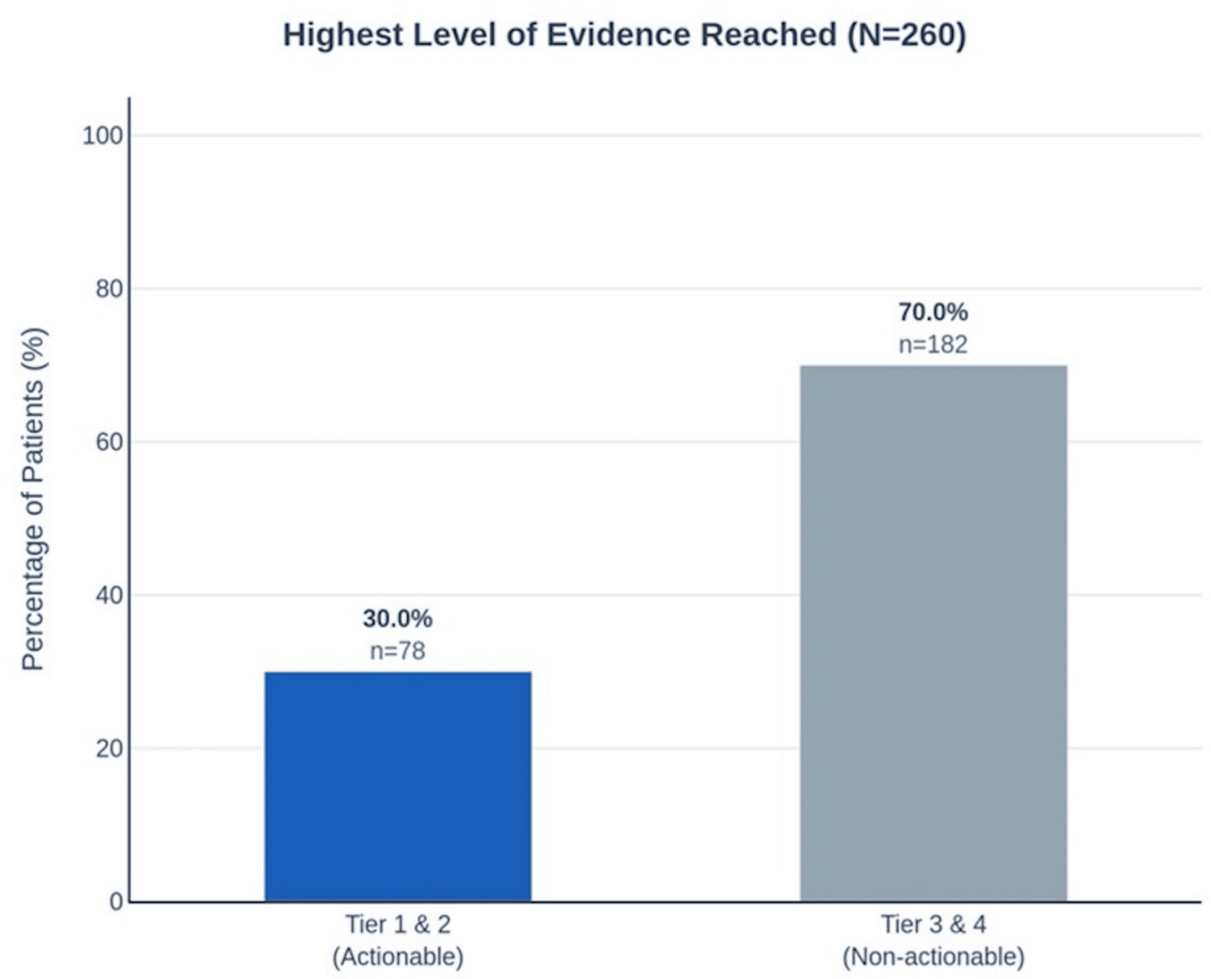
Evidence tier distribution. Distribution of the highest evidence tier reached across the patient cohort (N = 260). 30.0% of patients (n = 78) reached Tier 1 (Known Actionable) or Tier 2 (Likely Actionable), representing immediately actionable findings. The remaining 70.0% (n = 182) reached Tier 3 (Uncertain Significance) or Tier 4 (Likely Passenger) as their highest evidence tier; these patients still received output, including clinical trial eligibility listings surfaced through ontology expansion. Tier definitions are specified in Materials and Methods, Module 1. Tier 1: Known Actionable (United States Food and Drug Administration (FDA)-approved or OncoKB Level 1/2/R1/CIViC Level A evidence with matching cancer type). Tier 2: Likely Actionable (OncoKB Level 3A/3B, CIViC Level B, or COSMIC hotspot with recurrence ≥5). Tier 3: Uncertain Significance (predicted deleterious without clinical evidence). Tier 4: Likely Passenger (benign or tolerated predictions, no cancer gene relevance). The data chart was generated programmatically using standard data visualization libraries Python (Python Software Foundation, Wilmington, DE, USA).

**Figure 4:**
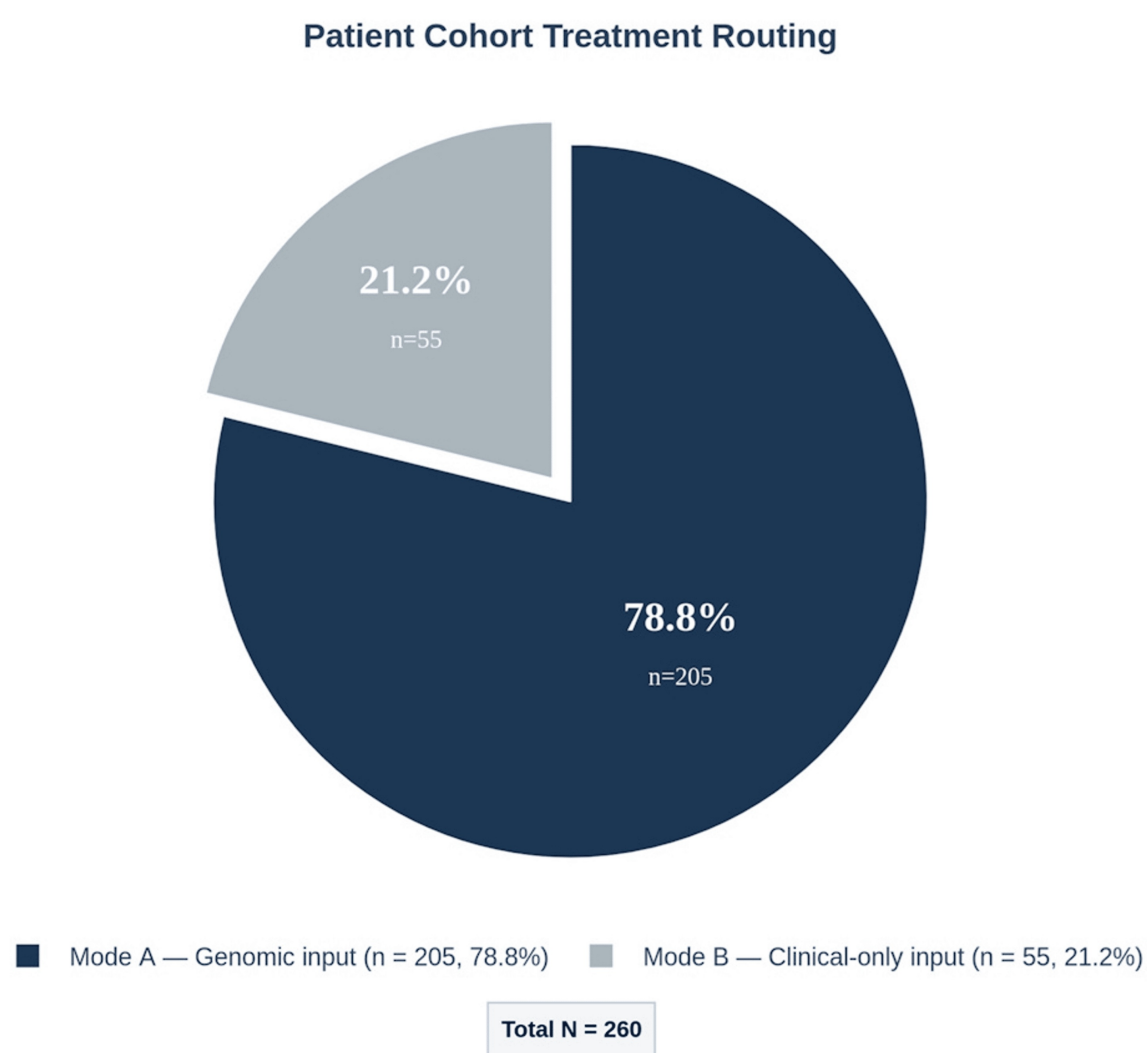
Patient routing. Distribution of patient routing across processing modes (N = 260). Mode A (biomarker-driven genomic match using patient-specific somatic mutations) was used for 78.8% of the cohort. Mode B (clinical-only fallback against curated subtype-level gene panels) was used for the remaining 21.2%, who were routed automatically because they lacked sufficient retained coding variants for genomic-mode processing. Both modes generate output spanning all four evidence tiers. The data chart was generated programmatically using standard data visualization libraries Python (Python Software Foundation, Wilmington, DE, USA).

At least one Tier 1 or Tier 2 drug match was identified in 78 of 260 patients (30.0%; 95% CI: 24.5-36.0%). These represent options with Food and Drug Administration (FDA) approval for the specific indication or strong genomic-matching evidence--the most immediately actionable tier (Figure 3). Matches derived from patient-specific somatic mutations were achieved in 205 of 260 patients (78.8%; 95% CI: 73.4-83.6%) (Figure 4). The remaining 55 patients (21.2%) were processed via clinical-only mode due to insufficient variant data, receiving matches based on commonly mutated genes for their sarcoma subtype.

The Tier 1/2 rate varied by histological subtype, consistent with known differences in molecular targetability. Subtypes with established targeted therapies showed higher rates than those lacking known druggable drivers. All 260 patients received at least one output at some evidence tier, with the non-Tier-1/2 patients receiving Tier 3 (uncertain significance/investigational) or Tier 4 (likely passenger/preclinical) matches and clinical trial eligibility listings. The Tier 1/2 rate is the primary endpoint because it represents immediately actionable findings.

**Figure 5:**
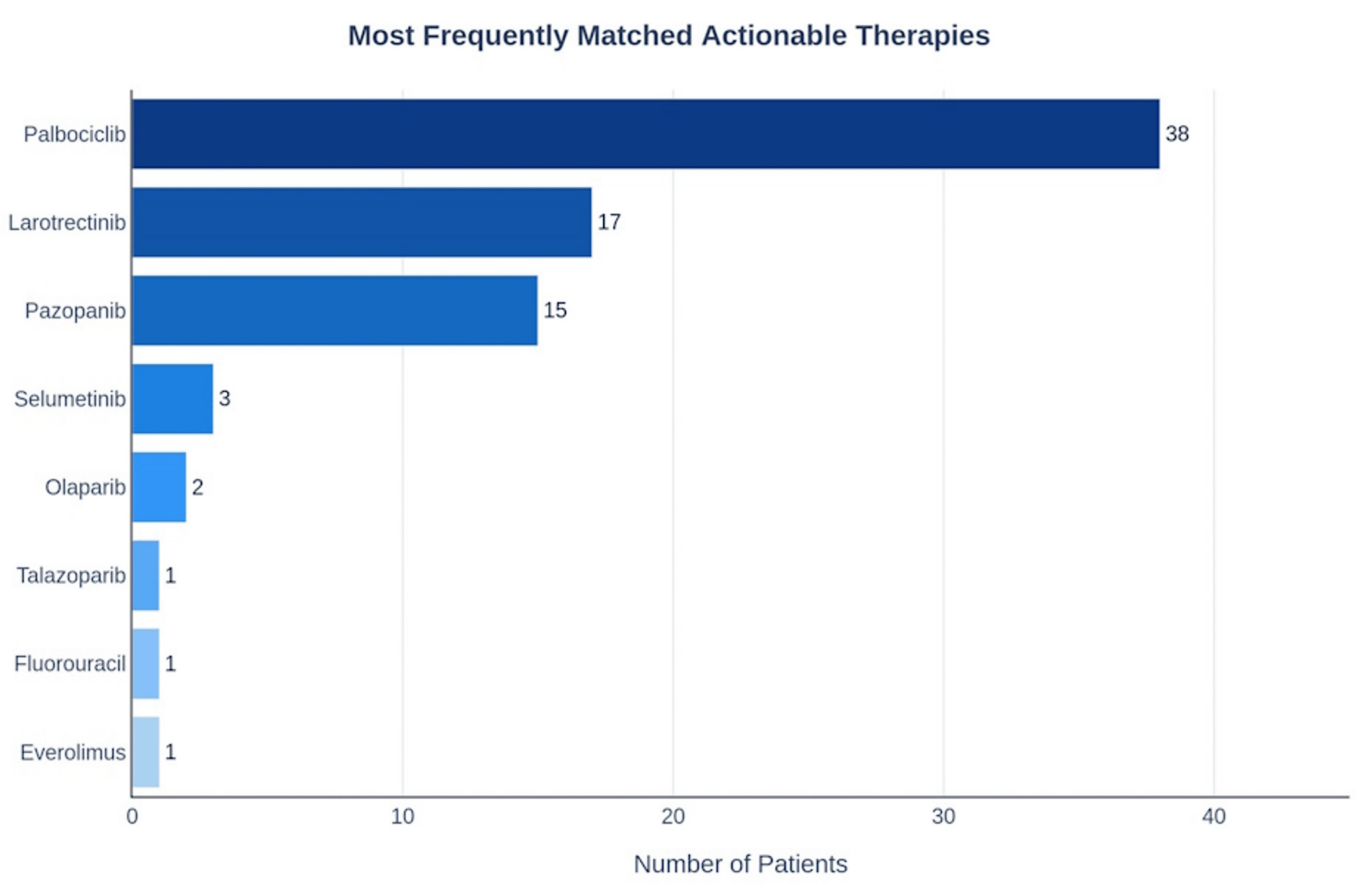
Most frequently matched therapies. Frequency distribution of Tier 1/2 actionable therapies matched to the 260-patient TCGA-SARC cohort. Counts represent unique patients for whom the named agent was the highest-tier match identified by the pipeline; a patient may also have lower-tier matches not shown. Palbociclib (cyclin-dependent kinase 4/6 (CDK4/6) inhibitor) was the most frequently matched agent (n = 38), reflecting the high prevalence of CDK4 amplification in dedifferentiated liposarcoma. Larotrectinib (n = 17) and pazopanib (n = 15) were the next most frequently matched. Full target and mechanism information for each agent is provided in Table 3. Counts shown sum to 78, which equals the number of patients reaching Tier 1 or Tier 2 (see Figure 3). Each patient is counted once, against their highest-tier matched agent. The data chart was generated programmatically using standard data visualization libraries Python (Python Software Foundation, Wilmington, DE, USA).

The most commonly identified actionable agents across the cohort are summarized in Table 3 and Figure 5. Palbociclib (cyclin-dependent kinase 4/6 (CDK4/6) inhibitor) was matched to 38 patients, consistent with the high prevalence of CDK4 amplification in dedifferentiated liposarcoma.

**Table 3:** Most frequently matched actionable therapeutic agents across the 260-patient TCGA-SARC cohort. The “Patients Matched (n)” column reports unique patients for whom the named agent was the highest-tier match identified by the pipeline; each patient is counted once. Counts sum to 78, equal to the total number of patients reaching Tier 1 or Tier 2 (see Figure 3). Tier 1: Known Actionable (United States Food and Drug Administration (FDA)-approved or strong clinical evidence). Tier 2: Likely Actionable (strong genomic match or hotspot). CDK: cyclin-dependent kinase; FDA: United States Food and Drug Administration; MEK: mitogen-activated protein kinase kinase; mTOR: mammalian target of rapamycin; NTRK: neurotrophic tyrosine receptor kinase; PARP: poly (ADP-ribose) polymerase; PDGFR: platelet-derived growth factor receptor; TCGA-SARC: The Cancer Genome Atlas Sarcoma cohort; VEGFR: vascular endothelial growth factor receptor.

Therapeutic agent	Primary target/mechanism	Max evidence tier	Patients matched (n)
Palbociclib	CDK4/CDK6	Tier 2	38
Larotrectinib	NTRK1/NTRK2/NTRK3	Tier 2	17
Pazopanib	VEGFR/PDGFR/c-KIT	Tier 2	15
Selumetinib	MEK1/MEK2	Tier 2	3
Olaparib	PARP1/PARP2	Tier 2	2
Talazoparib	PARP1/PARP2	Tier 2	1
Fluorouracil	Thymidylate synthase	Tier 2	1
Everolimus	mTOR	Tier 2	1

Representative clinical scenario

A representative clinical scenario was constructed to illustrate clinical-only mode operation. This case is a synthetic vignette constructed for demonstration purposes and does not represent any identifiable individual; no human subjects or clinical records were used. The case involves a middle-aged male with metastatic spindle cell sarcoma of the gluteal region with lung, retroperitoneal, and rib metastases, following surgical excision, radiation (progressive disease), targeted therapy, and salvage chemotherapy (partial metabolic response on positron emission tomography (PET) imaging); the case was processed via clinical-only mode.

The pipeline generated options spanning all four evidence tiers. Tier 1 and 2 options included drugs with genomic match evidence for sarcoma-associated molecular targets. Tier 3 options included recruiting clinical trials surfaced through ontology expansion--trials that would not have appeared under a direct "spindle cell sarcoma" search but became visible through "soft tissue sarcoma" and "sarcoma" parent queries. The pipeline also identified investigational modalities, including FAP-targeted radioligand therapy and immune checkpoint combinations, under active investigation for soft tissue sarcoma.

Each option was accompanied by a regulatory access pathway (standard prescription, clinical trial, off-label with NCCN compendium support, or compassionate use) and a next-step action item for clinician consideration. The output is structured as an evidence-ranked investigational option dossier for clinician review, not as a prescriptive recommendation.

Context-adaptive scoring and clamping verification

Deterministic weight clamping did not engage in any of the 260 standard evaluation runs across the TCGA-SARC cohort. This 0.0% trigger rate admits two non-exclusive interpretations: the LLM produced well-formed weight distributions for typical sarcoma scenarios, and/or the configured bounds were permissive relative to the LLM's natural output distribution for this class of inputs. The clamping mechanism is therefore presented as an engineering design pattern for clinical AI systems that require both adaptive reasoning and auditable bounds, rather than as a statistically verified safety net tested across a perturbation-based evaluation suite.

To verify functional engagement under edge-case inputs, a boundary condition test was executed using a constructed extreme clinical scenario. The system was instructed that the hypothetical patient demanded zero-toxicity exclusivity (designed to force safety = 1.0, all other dimensions = 0.0). The clamping system successfully intercepted the output, forced the safety component to its configured upper bound of 0.40, and redistributed weight across the remaining dimensions within their respective bounds. The re-normalized output maintained clinically reasonable proportions across all five scoring dimensions. This boundary condition test demonstrates that the mechanism activates as designed under a constructed edge case; systematic evaluation across a structured suite of prompt perturbations is designated as future work.

Compute performance

Total LLM API cost for the 260-patient cohort was $303.74 ($1.17 per patient). This figure reflects the interpretation and synthesis cost only and assumes that somatic variant data (MAF/VCF) are already available as input. It is therefore not directly comparable to end-to-end commercial genomic profiling services (for example, Foundation Medicine (Cambridge, MA, USA) or Tempus AI (Chicago, IL, USA)), which include DNA sequencing, Clinical Laboratory Improvement Amendments (CLIA)-certified laboratory processing, and board-certified pathologist sign-off in addition to interpretation [32]. The more relevant point of reference for the pipeline is the analyst time required for manual multi-database cross-referencing across DGIdb, CIViC, OncoKB, and ClinicalTrials.gov for each mutated gene--a process that typically requires hours of expert effort per case and is rarely performed systematically for rare sarcoma patients in community oncology settings [31]. In clinical deployment mode using a locally hosted open-source LLM, the per-case API cost is eliminated entirely, requiring only institutional compute resources.

RareCure is not CLIA-certified and is not a substitute for certified clinical genomic interpretation services. It is designed as a complementary rapid-screening tool that generates investigational option dossiers for clinician review. The practical significance of the low marginal interpretation cost is that repeated re-analysis becomes feasible as databases update and new trials open--a workflow previously constrained by manual curation bottlenecks.

## Discussion

Principal findings

RareCure identified at least one clinically actionable option in 30.0% of 260 soft tissue sarcoma patients (95% CI: 24.5-36.0%). Published actionable alteration rates in sarcoma genomic profiling range from 20% to 40%, depending on actionability definition [6], and the present results fall within this range, supporting the pipeline's tier classification validity. Because no comparable end-to-end system currently exists for rare tumor treatment discovery, a formal head-to-head comparison was not possible; the published actionable alteration ranges from independent genomic profiling studies [6] serves as the most appropriate external benchmark. The interpretation of the 30.0% rate with respect to knowledge-base overlap is addressed in a dedicated Discussion subsection below.

The 78.8% biomarker-driven matching rate demonstrates that the pipeline leveraged patient-specific mutations for the substantial majority of the cohort, moving beyond generic gene-cancer associations. The remaining 21.2% processed via clinical-only mode highlight the real-world challenge of incomplete genomic data--a challenge the pipeline accommodates through its dual-mode architecture.

Dual deployment architecture

The model-agnostic design serves two distinct use cases. For research applications using de-identified public datasets, cloud-hosted LLMs offer convenience and state-of-the-art performance. For clinical deployment involving protected health information, locally hosted open-source models ensure that no patient data traverses external networks. The pipeline logic, module interfaces, and scoring methodology are identical in both configurations; only the LLM endpoint differs. This separation means that the validation results reported here (using Claude on public TCGA data) are designed to be transferable to institutional deployment environments (using local Llama on institutional data), as the scoring methodology is model-agnostic and the deterministic clamping bounds are independent of the underlying LLM. Formal cross-model equivalence testing is designated as future work.

Interpreting the 30% actionability rate

The 30.0% Tier 1/2 rate does not mean 70% lack options. Rather, 70% lack options at the highest evidence tiers. Many had Tier 3 or Tier 4 matches representing viable pathways through trial enrollment or compassionate use. The pipeline presents all tiers; the Tier 1/2 rate is the primary endpoint because these findings are most likely to result in immediate therapeutic action.

For the 78 patients with Tier 1/2 matches, the pipeline surfaced options that would otherwise require manual cross-referencing of DGIdb, CIViC, OncoKB, and ClinicalTrials.gov for each mutated gene--a process that rarely occurs for rare sarcoma patients in community oncology settings.

Validation cohort and knowledge-base overlap

OncoKB's actionability classifications are curated from clinical evidence that includes genomic studies contemporaneous with or downstream of the TCGA effort. When the pipeline queries OncoKB to annotate TCGA-SARC variants, partial overlap therefore exists between the validation cohort and the data informing the knowledge base. This circularity is inherent to any system applying curated oncology knowledge bases to well-characterized public cohorts, and it affects all evidence-tier assignments reported here. Prospective validation on newly sequenced patients, or retrospective validation on post-TCGA cohorts such as the American Association for Cancer Research (AACR) Project Genomics Evidence Neoplasia Information Exchange (GENIE) sarcoma subset, would eliminate this overlap and is designated as the primary external validation pathway for ongoing work. The 30.0% actionability rate reported in this study should therefore be interpreted as consistent with published sarcoma benchmarks rather than as an independent performance estimate.

Clamping as an engineering design pattern

The 0.0% trigger rate in standard evaluation, combined with successful engagement under a constructed boundary condition test, should be interpreted as functional verification that the clamping handler engages at the edge of its operating envelope, not as statistical validation of its safety properties across the input distribution. Verification here refers to confirming that the implemented mechanism behaves as specified under a defined test input; validation would additionally require demonstrating that the mechanism achieves its intended clinical objective across a representative range of real patient scenarios, which has not been done. The mechanism is, therefore, offered as an engineering pattern for clinical AI systems that require both adaptive reasoning and auditable bounds: LLM-guided weight generation constrained by deterministic, version-controlled limits. The configured bounds (for example, evidence strength never below 5% or above 60%) can be adjusted by institutions to reflect local clinical protocols or governance requirements. This design pattern--LLM reasoning within deterministic guardrails--may be applicable to other clinical AI applications where adaptive behavior must coexist with regulatory auditability.

Nothing in this manuscript should be construed as medical advice. The methods, tools, and outputs described are intended to support research exploration and to generate investigational option dossiers for review by qualified healthcare professionals.

Interpretation cost and access

At $1.17 per patient in research mode and zero incremental LLM cost in local deployment mode, multi-database investigational treatment-option screening becomes achievable for any research institution with existing genomic data. The total cohort cost of $303.74 covers interpretation and synthesis across variant annotation, drug-gene matching, trial matching, and literature evidence synthesis. This does not include DNA sequencing or certified clinical interpretation, which remain prerequisites for any clinical application. The practical benefit of low marginal interpretation cost is enabling workflows previously infeasible at scale: screening all patients at a sarcoma center, providing analysis in resource-limited research settings, or longitudinal re-analysis as new drugs and trials emerge.

Broader impact

This work contributes to the effort to accelerate precision oncology research for underserved cancer populations. Rare cancer patients face a structural inequity: their tumors are too uncommon to attract dedicated drug development programs, yet they require the same precision-medicine infrastructure as common cancers. By open-sourcing an end-to-end pipeline that performs multi-database treatment matching at negligible marginal interpretation cost, this research provides a tool that any research institution or cancer center can adopt without licensing barriers.

The methodology is applicable across research institutions. Because RareCure relies exclusively on public databases, open-source models, and standard data formats (MAF/VCF, Fast Healthcare Interoperability Resources (FHIR)-compatible clinical profiles), adoption requires no proprietary infrastructure. The dual deployment architecture--cloud for research, local for protected data--supports institutions with varying computational resources and regulatory obligations.

The ongoing research agenda, including module-level ablation, external validation on post-TCGA cohorts, neoantigen prediction validation, combination therapy modeling, and expansion to additional rare cancer types, is designed to progressively strengthen the evidence base before any clinical application is contemplated.

Limitations

This study has several limitations, primarily related to retrospective validation, architectural scope, and regulatory status. Retrospective design and cohort bias: Because the TCGA-SARC cohort comprises patients treated before many identified therapies were available, outputs cannot be evaluated against survival or response outcomes. Furthermore, the cohort originates predominantly from U.S. academic centers and may not reflect global genomic diversity. Prospective evaluation by a molecular tumor board on newly sequenced patients is required. Knowledge-base circularity: As discussed in the dedicated Discussion subsection, partial overlap exists between the genomic profiles in the TCGA validation cohort and the historical evidence used to construct OncoKB's actionability classifications. External validation on post-TCGA cohorts, such as the AACR Project Genomics Evidence Neoplasia Information Exchange (GENIE) sarcoma subset, is designated as the primary remediation pathway. Validation gaps in modular components: The pipeline is evaluated end-to-end, but module-level ablation analysis isolating the marginal contribution of specific components--for example, the exact quantitative impact of ontology expansion on trial discovery or the effect of context-adaptive scoring versus fixed weights--was not performed. Additionally, while the neoantigen prediction module was successfully implemented to demonstrate architectural compatibility with both personalized human leukocyte antigen (HLA) typing and HLA supertypes, no neoantigen-specific outputs are reported in this study's results, and batch-scale validation against experimental immunopeptidomics remains designated as future work. Clamping behavior was verified through a single boundary condition test rather than through a structured perturbation suite. Clinical and biological scope: The system evaluates single therapeutic agents and does not model combination regimens, overlapping toxicities, drug-drug interactions, or patient-specific organ function and comorbidities beyond explicit prompt inputs. Outputs are also dependent on point-in-time database refreshes and will drift out of currency as new trials open and new evidence is curated. The reported cost figures reflect interpretation and synthesis only and do not include upstream DNA sequencing or certified clinical interpretation, which remain prerequisites for any clinical use. Algorithmic non-determinism: While deterministic clamping strictly enforces output bounds, the underlying large language model (LLM) introduces marginal scoring variations between runs within those limits. All reported results utilized Claude 3.5 Sonnet; cross-model equivalence across alternative LLM providers--including the locally hosted open-source models envisioned for clinical deployment mode--has not yet been formally established. Regulatory status: RareCure is an investigational research prototype. It is not Clinical Laboratory Improvement Amendments (CLIA)-certified, is not a regulated medical device, and generates investigational option dossiers for clinician review rather than prescriptive treatment plans. It is strictly not designed or validated for direct patient care applications.

## Conclusions

RareCure is an open-source artificial intelligence pipeline that automates treatment discovery for rare solid tumors within a single integrated workflow. In the TCGA-SARC cohort (N = 260), Tier 1 or Tier 2 options were identified in 30.0% of patients (95% CI: 24.5-36.0%), a rate consistent with the range reported in independent sarcoma genomic profiling studies as discussed above. Biomarker-driven matching from patient-specific mutations was achieved in 78.8% of cases. The per-patient interpretation cost of $1.17 in research mode, and zero incremental LLM cost in local deployment mode, supports repeated re-analysis as databases and clinical trial listings update--a workflow previously constrained by manual curation bottlenecks. The context-adaptive scoring system with deterministic clamping provides an engineering pattern for clinical AI: adaptive LLM reasoning within deterministically auditable bounds. The 0.0% standard trigger rate, combined with successful engagement under an extreme boundary condition test, provides functional verification that the mechanism engages as specified; it does not substitute for validation across a representative range of real patient scenarios.

For rare cancer patients whose oncologists have exhausted standard options, systematic and repeatable screening across variant databases, clinical trial registries, and indexed literature is a prerequisite for identifying options that would otherwise remain hidden across four disconnected databases. The findings presented should not be interpreted as clinical recommendations; they demonstrate that an integrated, automated pipeline can produce literature-concordant actionability rates at low marginal interpretation cost. Further validation--including module-level ablation, external cohort testing on post-TCGA sarcoma data (for example, the AACR Project GENIE sarcoma subset), prospective molecular tumor board comparison, batch-scale neoantigen validation, systematic perturbation-based evaluation of the clamping mechanism, and cross-model equivalence testing--is required before any clinical application and forms the core of the ongoing research agenda.
